# Intraductal papillary neoplasm of intrahepatic bile ducts complicated by chronic disseminated intravascular coagulation and thrombosis: A case report

**DOI:** 10.1097/MD.0000000000024454

**Published:** 2021-02-05

**Authors:** Ming Xiao, Aijun Sun, Fan Yu, Ying Xiao, Lihong Li, Dongyan Shen, Canhong Xiang, Jiahong Dong

**Affiliations:** a Center of Hepatopancreatobiliary Diseases; b Department of Hematology; c Department of Pathology; d Department of Cardiac Surgery, Beijing; e Department of Hepatobiliary Surgery, Zhucheng People's Hospital, Zhucheng, China.

**Keywords:** anemia, chronic disseminated intravascular coagulation, intraductal papillary neoplasm of the bile ducts, thrombosis, trousseau's syndrome

## Abstract

**Rationale:**

Intraductal papillary neoplasm of the bile ducts (IPNB) is a relatively rare tumor that is clinically characterized by digestive symptoms. The concurrent occurrence of chronic disseminated intravascular coagulation (DIC) with thrombosis is an extremely rare combination, reported in patients with IPNB. The clinical features of chronic DIC include microangiopathic hemolytic anemia, thrombocytopenia, and hypofibrinogenemia. Here, we report the case of a mucin-producing IPNB patient with hematological abnormalities.

**Patient concerns:**

A 58-year-old male patient suffered from abdominal distension for more than 2 months with obstructive jaundice appearance. Abdominal contrast-enhanced computed tomography and magnetic resonance cholangiopancreatography showed a neoplasm in the right hepatic lobe. Multiple intravascular fillings were found in the inferior vena cava, pulmonary artery, and right atrium. Anemia and hypofibrinogenemia were discovered through routine laboratory tests. The count of platelets began to decline 25 days after admission, while 1 month after hospitalization, the patient developed abdominal pain, fever, and shock.

**Diagnosis:**

Pathological examination demonstrated IPNB with a part of high-grade intraepithelial neoplasia. Cardiac and inferior vena cava emboli were diagnosed as thrombi without neoplastic cells. Immunohistochemically, tumor cells were positive for Vimentin (mesenchyme), CK7, CK19, MUC-1, MUC-5AC, MUC-6, S-100p (focal), Ki-67 (12%), and negative for Inhibin-α, ER, CK20, CEA, and MUC-2. Additionally, immunohistochemistry indicated that IPNB was a mucus-secretion gastric type. The laboratory tests confirmed the presence of chronic DIC.

**Interventions:**

The patient was given anticoagulant therapy before hepatectomy and right atrium thrombectomy was performed under cardiopulmonary bypass.

**Outcomes:**

After anticoagulant therapy, the levels of hemoglobin, platelet, and fibrinogen of the patient returned to normal. Hepatectomy and thrombus removal was successfully performed. Then, the patient was discharged 12 days after the operation. After 12 months of follow-up, the patient recovered well without any hematologic abnormalities and no signs of tumor recurrence were observed.

**Lessons:**

IPNB may cause hematological complications, which can be easily misdiagnosed. It is essential to pay particular attention to the hematological abnormalities of patients with IPNB. Early detection and differential diagnosis of chronic DIC and thrombosis are necessary. We note that anticoagulant therapy coupled with surgery is an effective strategy to treat these complications.

## 1 Introduction

Intraductal papillary neoplasm of the bile ducts (IPNB) is a kind of epithelial tumor with malignant potential that undergoes papillary or villous growth in the bile duct lumen. It is characterized by the secretion of a large amount of mucus that causes cholangiectasis.^[1,2]^ Up to 44% of patients present with intraluminal mucin. IPNB was once identified as mucin-producing cholangiocarcinoma, mucin-secreting biliary tumor, or bile duct papillary myxoma.^[2,3]^ Moreover, it has been named as IPNB according to the classification of tumors of the digestive system of WHO since 2010. In East Asia, the incidence rate of Intraductal papillary neoplasm (IPNs) is estimated at 10% to 38% among all biliary tumors.^[1]^ Notably, abdominal pain, cholangitis, and jaundice are the most prevalent clinical manifestations associated with 14% to 42% of patients, but hematologic manifestations have not been reported.^[3]^ In addition, Trousseau syndrome is ascribed to multiple manifestations of coagulopathy, namely, pathological hypercoagulability, chronic disseminated intravascular coagulation (DIC), and thrombosis in patients with carcinoma.^[4,5]^ On the other hand, mucin-positive carcinoma is often correlated with Trousseau syndrome. However, the benign mucin-producing disease can also develop hypercoagulability which is similar to Trousseau syndrome.^[6]^ Given this background, we herein report a case of the coexistence of IPNB and hematologic abnormalities.

## 2 Case report

**Figure 1:**
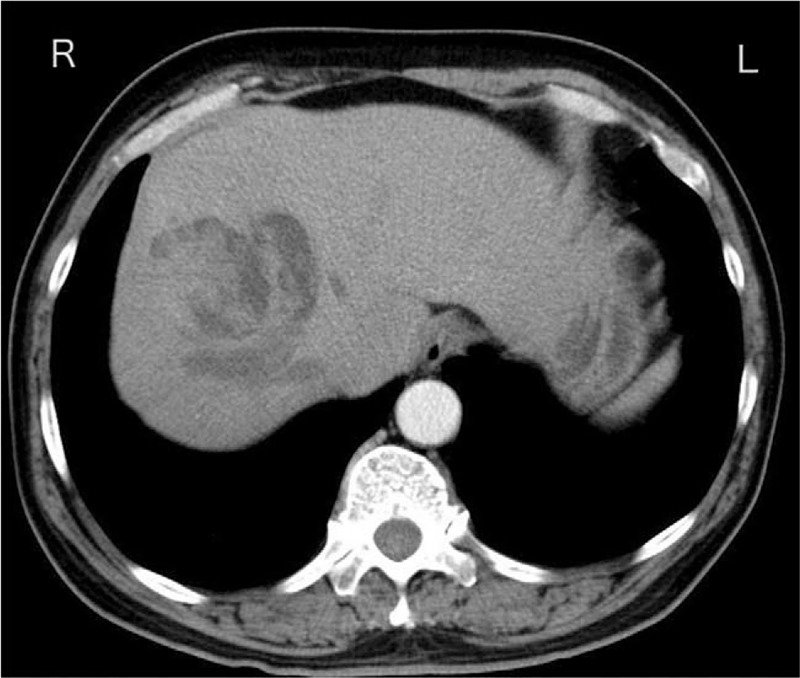
Irregular lump shadow in the bile duct with dilation of the surrounding bile duct and mild enhancement of the lump in the arterial phase.

**Figure 2:**
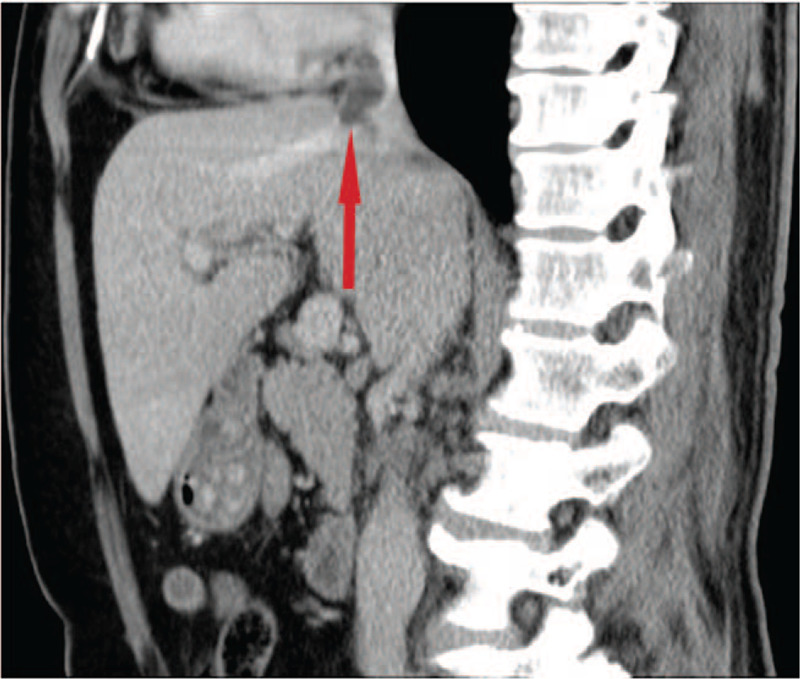
Filling defect observed in the inferior vena cava (↑).

**Figure 3:**
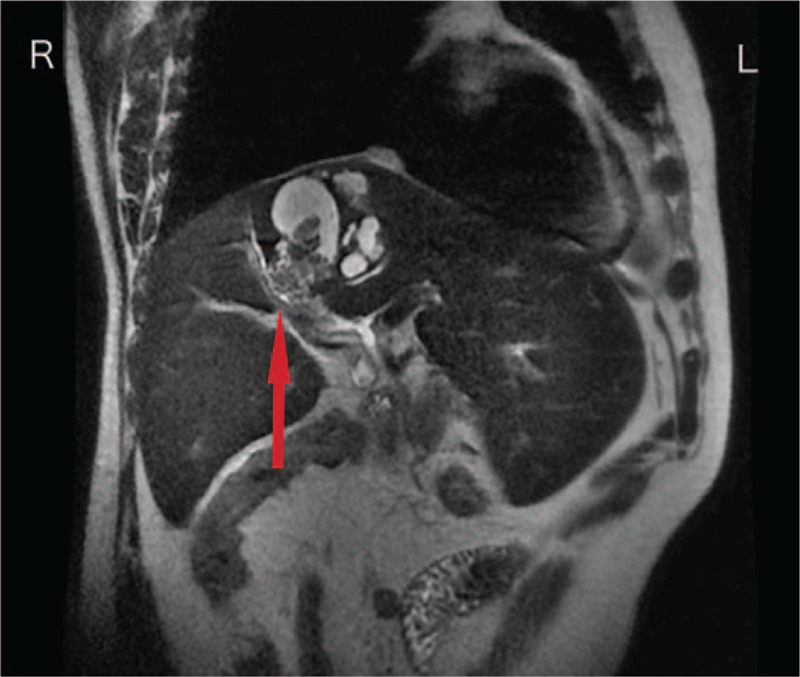
MRCP showed a “cauliflower-like” lump shadow (↑).

**Figure 4:**
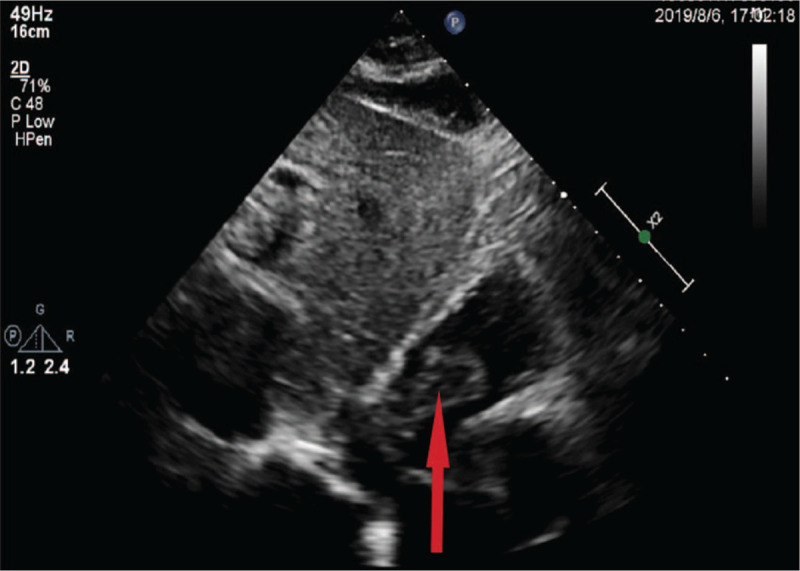
An echocardiogram showed an irregular mass shadow in the right atrium (↑).

A 58-year-old man was admitted to our hospital due to abdominal distension for the last 2 months, accompanied by yellow staining of skin and sclera and darker color of urine for 13 days. Based on the physical examination, we observed no abnormalities except for an icteric appearance. Additionally, an irregular lump was observed in the right lobe of the liver with unclear boundaries on abdominal contrast-enhanced computed tomography. Multiple tubular and patchy images appeared around the lesion, while the dilated bile duct was inspected at the distal end of the lesion. We identified that the solid components of the lesion were slightly enhanced with an average computed tomography value of about 66 HU (Fig. 1). We also viewed filling defects in the inferior vena cava with no clear enhancement (Fig. 2). Magnetic resonance cholangiopancreatography revealed that the right hepatic intrahepatic bile ducts were dilated to varying degrees, some of which were cystic. In the dilated bile ducts, there was an irregular and slightly longer T2 signal lump shadow, some of which were “cauliflower-like,” whereas the partial liver margin was slightly sunken (Fig. 3). Using an echocardiogram, we discovered an irregular mass-like echo filling in the right atrium, with divided lobes visible (Fig. 4). In particular, the larger lobe was approximately 47 mm × 18 mm in size, starting from the entrance of the inferior vena cava. We also uncovered small filling defects in the posterior pulmonary artery in the left and right upper lobes using computed tomographic pulmonary angiography (CTPA). As the patient had space-occupying lesions in the inferior vena cava and atrium along with pulmonary embolism, the Caprini grade was high-risk. Without bleeding tendency, low molecular weight heparin (LMWH) was used for thromboembolic prophylaxis twice a day (100 IU/Kg). In addition to the mucus secreted by the tumor retained in the bile duct, there may also be a bile duct tumor thrombus that caused obstruction and then formed cystic dilatation, leading to jaundice due to the obstruction of bile (total bilirubin 103.56 umol/L; direct bilirubin 54.41 umol/L; urine bilirubin 17 umol/L). The tumor was close to the right hepatic vein, with probability of invading the blood vessel. Our preliminary diagnosis of the tumor was considered to be malignant with vascular and atrium tumor thrombi (CA-199 174.76 U/mL), and thus surgery was planned. However, hematologic abnormalities co-existed.

Here, the laboratory results showed moderate anemia categorized as normocytic and normochromic (red blood cells, 2.11 × 10^12^ /L; hemoglobin 68 g/L; mean corpuscular volume 90 fL; and mean corpuscular hemoglobin 32.20 pg/Cell). We observed that several coagulation indexes were slightly elevated, whereas fibrinogen and high D-dimer were decreased (prothrombin time 13.5 seconds; prothrombin time ratio 1.21; fibrinogen 0.97 g/L; D-dimer 7.15 mg/LFEU). Notably, transfusion of suspended erythrocytes failed to correct anemia. We further performed blood tests to establish the potential cause of the anemia. Direct evidence of erythrocyte destruction (indirect bilirubin, 49.15 umol/L; lactate dehydrogenase 460 U/L; peripheral morphological analysis of blood cells presented approximately 3% of broken erythrocytes) and indirect evidence (significantly elevated reticulocytes (%), 16.31%) were both present, thereby the diagnosis of hemolytic anemia was definite in spite of unclear pathogenesis. Accompanied by decreased platelets (from 176 × 10^9^/L to 116 × 10^9^/L), autoimmune hemolytic anemia combined with immune thrombocytopenia (Evans syndrome) were not excluded. Subsequently, the patient was empirically given glucocorticoid for 6 days (0.1 mg/Kg body weight). After 8 days, the patient presented no improvement of anemia with a further decreased count of platelets (64 × 10^9^/L), and thus the patient was immediately transferred to the Department of Hematology for further treatment. Consequently, the autoimmune antibody, fluorescently labeled modified aerolysin (FLAER), CD55\CD59 expression, and direct antiglobulin (Coombs) tests were not abnormal, which ruled out paroxysmal nocturnal hemoglobinuria (PNH) and autoimmune hemolytic anemia. Regarding that, the patient had no fever (leukocyte 6.66 × 10^9^/L; blood culture: no bacterial growth was observed), renal insufficiency, and neurological symptoms, and at the same time, the activity of ADAMTS13 was 68% and ADAMTS13 antibody was negative, thrombotic thrombocytopenic purpura (TTP) was also excluded. Moreover, 1 month after admission, the patient developed the following symptoms including sudden abdominal pain, fever, and decreased blood pressure (76/55 mmHg), together with increased infection indicators and deterioration of coagulation function [total bilirubin 690.4 umol/L, direct bilirubin 545.5 umol/L, C-reactive protein 74.17 mg/L, leukocyte 16.00 × 10^9^/L (NEUT% 96.50%), platelets 54.00 × 10^9^ /L, prothrombin time 16.5 seconds, prothrombin time ratio 1.47, and fibrinogen 1.63 g/L]. Acute cholangitis followed by shock were diagnosed, and decompression of the biliary tract was performed immediately. Thereafter, the patient was transferred to the intensive care unit (ICU) for supportive treatment, namely antimicrobial therapy and plasma and blood transfusion, but the anemia did not improve significantly. Due to the prolonged prothrombin time, LMHW was not given. Then, after 4 days, the patient was transferred to the general ward in a stable condition. At this time, anemia, thrombocytopenia, and hypofibrinogenemia still existed (hemoglobin 56 g/L; platelets 67 × 10^9^/L; fibrinogen 1.27 g/L). After a multidisciplinary team discussion, the probability of chronic DIC was high. After 20 days of oral rivaroxaban treatment, the patient was checked again, and the platelet and fibrinogen levels had returned to normal (platelets 293 × 10^9^/L, fibrinogen 3.87 g/L), while hemoglobin was significantly improved (122 g/L). Therefore, right trilobe hepatic resection plus thrombus removal from the inferior vena cava and atrium was performed.

**Figure 5:**
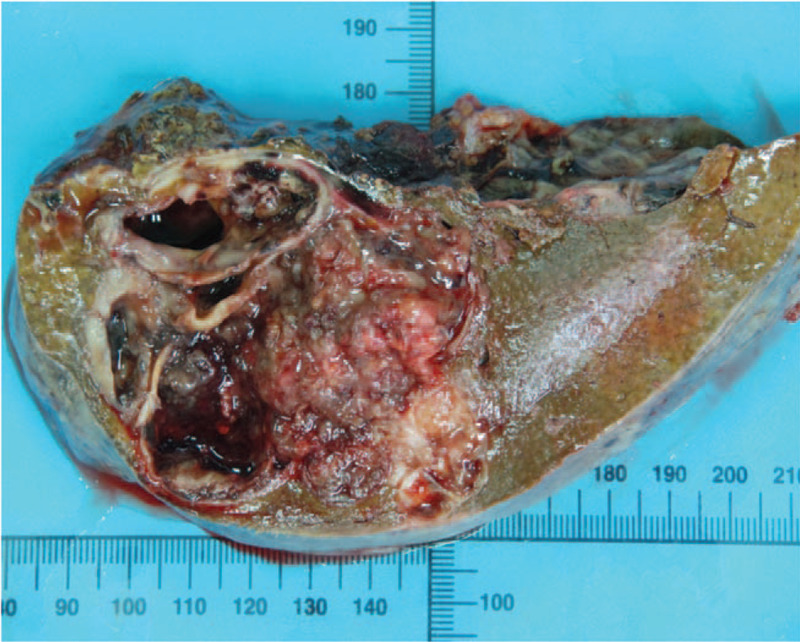
The gross specimen showed that the tumor was cystic, and mucus was observed in the cyst.

**Figure 6:**
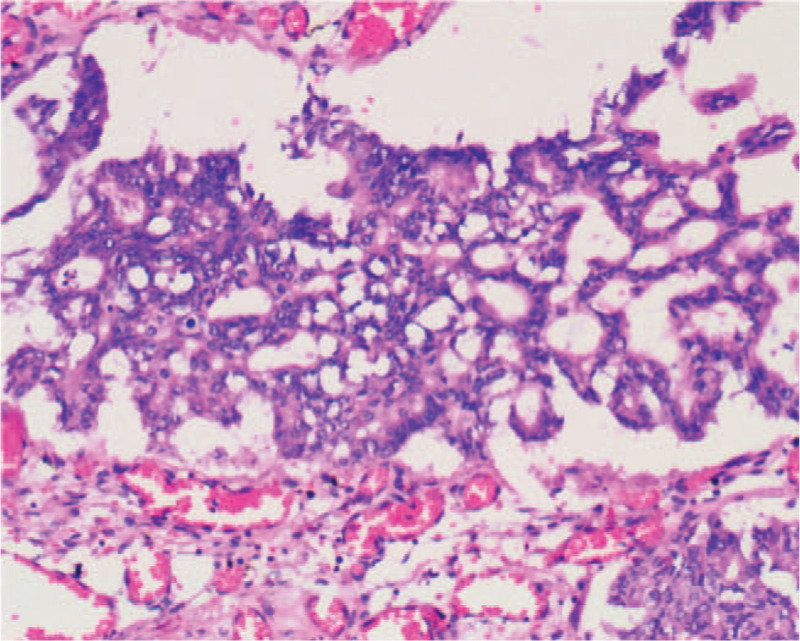
High-grade intraepithelial neoplasia.

**Figure 7:**
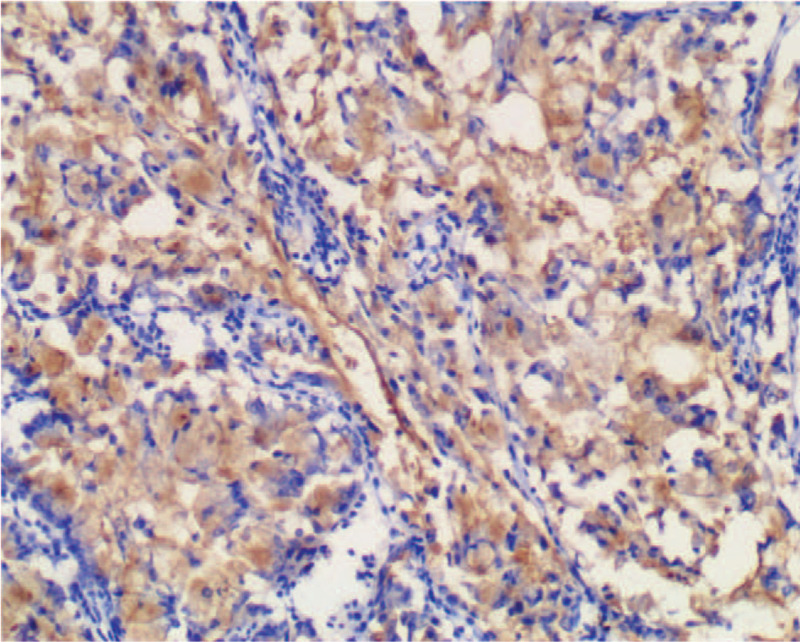
Immunohistochemical staining MUC-1 expression was positive.

**Figure 8:**
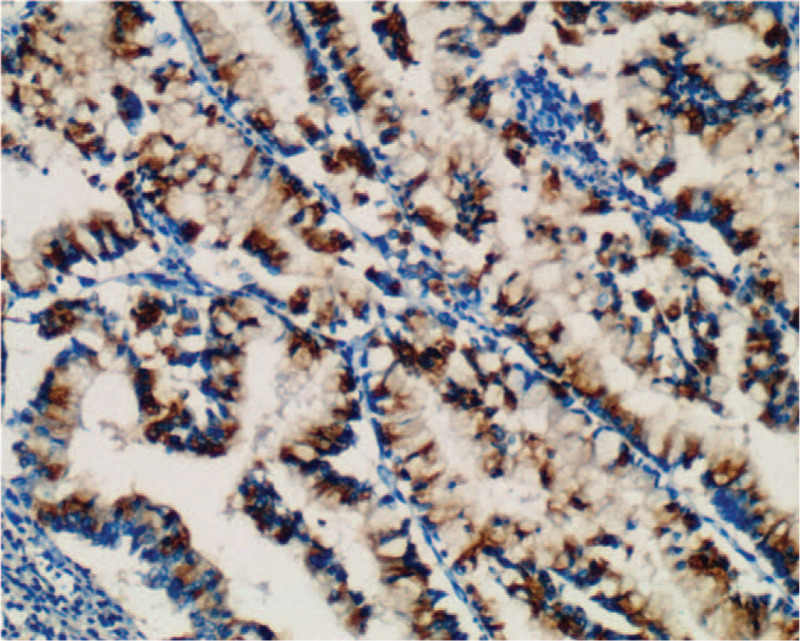
Immunohistochemical staining MUC-5AC expression was positive.

**Figure 9:**
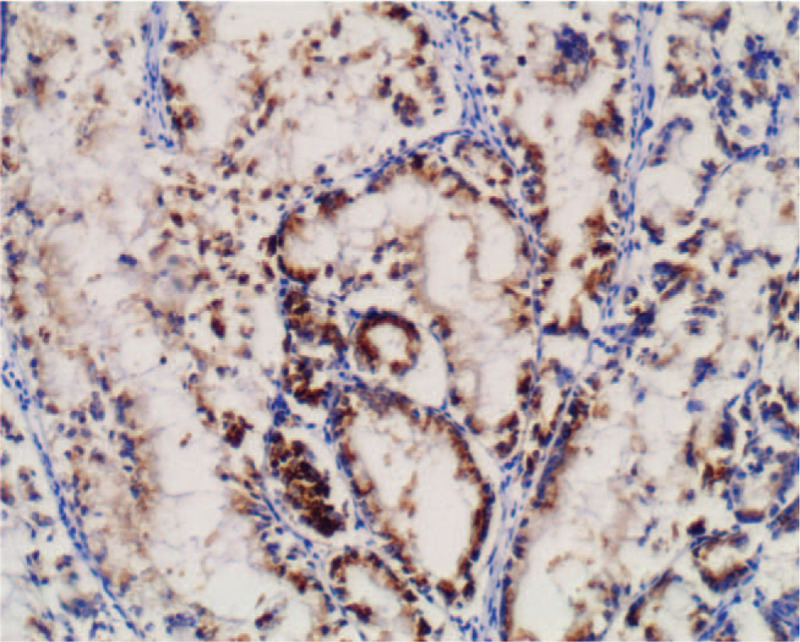
Immunohistochemical staining MUC-6 expression was positive.

Macroscopically, the intrahepatic cystic nodular lump presented size of 7 cm × 6 cm × 5.5 cm, in which the cut surface was multilocular cystic. We observed no solid area. Furthermore, the diameters of the cysts were between 1.5 and 5 cm, while the inner part of the wall of the cyst was attached with gray-red jelly-like substance and mucus (Fig. 5). Histologically, intraductal papillary neoplasms of the intrahepatic bile duct showed a part of high-grade intraepithelial neoplasia, without invading the liver capsule (Fig. 6). We established that cardiac and inferior vena cava emboli were fibrinoid mesh without neoplasms and thus were classified as thrombus. Conversely, biliary emboli were tumor tissue accompanied by hemorrhage and necrosis. Tumor immunohistochemical analyses were presented as follows: Inhibin-α (-), ER (-), Vimentin (mesenchyme +), CK7 (+), CK19 (+), CK20 (−), CEA (−), MUC-1 (+) (Fig. 7), MUC-5AC (+) (Fig. 8), MUC-6 (+) (Fig. 9), MUC-2 (−), S-100p (focal+), and Ki-67 (12% +). Finally, the patient recovered well without complications, and was discharged 12 days after surgery, and followed up for 12 months. No signs of tumor recurrence were observed.

## 3 Discussion

This case presents an intraductal papillary neoplasm of the bile duct (IPNB) patient with extremely rare complications. After anticoagulant therapy, we noted that hematologic abnormalities returned to normal. We subsequently performed the operation, whereby IPNB was histopathologically confirmed to secrete mucus, simultaneously suggesting the occurrence of rare hematologic complications.

DIC is defined as the activation of coagulation factors and platelets under the action of some pathogenic factors characterized by the occurrence of thrombosis, bleeding, or both. Empirical studies have established that the following conditions including trauma, obstetric surgery, sepsis, or cancer can trigger DIC.^[7]^ According to a retrospective study of 1117 patients with distinct solid tumors, DIC was diagnosed in 6.8% patients. More than half of DIC patients were diagnosed as adenocarcinoma. 3.9% of DIC patients had tumors located in the liver.^[8]^ Additionally, compared to acute DIC, chronic DIC may last several months asymptomatically and only manifest as occult laboratory abnormalities of excess thrombin generation, therefore making it extremely difficult to be diagnosed. In this case study, DIC was not initially diagnostic, because the presenting hemostatic markers were not typically based on the diagnostic scores of the Japanese Ministry of Health and Welfare (JMHW) or the International Society of Thrombosis and Hemostasis (ISTH). However, a persistent search for a reasonable etiology of anemia ultimately led to chronic DIC. Excluding other factors inducing DIC, such as sepsis, leukemia, trauma, and aneurysm, we identified that solid tumor was only possible due to DIC. Currently, there is no uniform scoring standard for tumor-related DIC in the world. However, some scholars and expert consensus suggest using fibrinogen and D-dimer to evaluate tumor-related DIC.^[9,10]^ Simultaneously, it is also recommended to use a decline in the platelet count (for example, more than 30%), rather than the absolute value, as the evaluation index. In general, a decrease in platelet count or a clear downward trend is one of the sensitive indicators of DIC, and its incidence can be as high as 98%.^[11]^ Previous studies have reported that a moderate or significant decrease in platelet count is also predominant in patients with tumor-related DIC.^[9]^ Another study by Kawasugi et al demonstrated that platelet counts, as well as fibrinogen in patients with solid tumors, were significantly lower in DIC patients compared to non-DIC patients (95.6% vs 55.6%, 21.7% vs 3.1%, respectively).^[12]^ Numerous reports have concluded that DIC can cause microangiopathic hemolytic anemia.^[9,10]^ This is because when circulating erythrocytes flow through the mesh composed of fibrin filaments, they often adhere to the fibrin filaments, coupled with the continuous impact of blood flow, causing erythrocytes to rupture. A second plausible explanation is that when the erythrocytes pass through the blood vessel at the microcirculation barrier, they can be squeezed out of the blood vessel through the gap between the vascular endothelial cells, and thus appears to be broken. Our patient's fibrinogen was constantly low, accompanied by anemia, increased D-dimer, decreased platelets, and coagulation disorder, hence presenting thrombosis as the primary manifestation for 1.5 months, which was consistent with the presentation of chronic DIC.

Furthermore, in this case, the emboli in the inferior vena cava and atrial were histologically confirmed as thrombosis after surgery. Therefore, it was inferred that the patient exhibited pathological hypercoagulability similar to Trousseau syndrome. Trousseau syndrome refers to the secondary hypercoagulable state or thrombosis in patients with malignant tumors that was firstly reported by Armand Trousseau in 1865.^[4]^ In clinical practice, venous thrombosis is prevalent, while arterial thrombosis is relatively rare.^[13]^ The specific underlying mechanism is complex and it may be associated with a series of mechanisms, including tumor mucus protein, tissue factors released by the tumor, tumor hypoxia, tumor-associated cysteine proteases, and oncogene activation.^[4,13]^ Of note, Trousseau syndrome is more dominant in mucus-secreting adenocarcinoma. This may be attributed to the fact that cancer cells often up-regulate the expression of multiple mucin polypeptides, for example, MUC1, MUC2, MUC5AC, MUC4, and MUC16.^[14–16]^ Multiple studies have revealed that a mixture of abnormal mucins can be released into the blood by cancer cells in large quantities, exhibiting the effect of promoting blood coagulation.^[4,16–18]^ A study by Wahrenbrock et al demonstrated that circulating mucin interacts with leukocyte L-selectin and platelet P-selectin to cause Trousseau syndrome through animal experiments.^[18]^ It is important to note that there are reports which have uncovered some benign diseases that produce mucins, such as adenomyosis, which may also lead to a hypercoagulable state and thrombosis, thereby resulting in cerebral infarction.^[6]^ The IPNB tumor in our patient secreted a large amount of mucus, with immunohistochemistry MUC-1, MUC-6, and MUC-5AC (+), which might be the cause of thrombosis in this patient. Studies have shown that heparin or LMWH can be used to treat tumor-related thrombosis.^[4]^ However, this patient required surgery in order to remove the thrombus due to the large thrombus volume. In addition, Sack et al reported that patients with Trousseau syndrome can also be complicated with chronic DIC.^[5]^ This finding implies that the coexistence of thrombosis and chronic DIC is not accidental. Thus, it may be ascribed to a tumor-related hematologic disorder based on the above analysis.

At the initial stage of treatment, LMWH was aimed at preventing further thrombogenesis. After treatment with rivaroxaban, the levels of fibrinogen, hemoglobin, and platelet of our patient returned to normal. It is known that mucinous tumors, such as pancreatic, gastric, and ovarian tumors are capable of secreting enzymes that have abilities activating coagulation factor X. Remarkably, LMWH and rivaroxaban inhibited the coagulation factor Xa (FXa), hence preventing thrombosis, such that the microcirculation was improved, while the secondary consumption of platelets was alleviated.^[4]^ Despite that LMWH exhibits an extremely low possibility of 0.06% to bring about heparin-induced thrombocytopenia (HIT), the decrease of platelets cannot be explained by HIT. We found that the platelets of our patient did not decrease during the first 25 days of using LMWH, whereas the onset of HIT occurred between 5 and 10 days after heparin treatment. Besides, a low 4T score of this patient (3 points) showed a negative predictive result of HIT.^[19,20]^ Therefore, clinically, when patients with tumors have unexplained anemia outside the blood and immune system, thrombocytopenia, and hypofibrinogenemia, it is necessary to be vigilant about the occurrence of tumor-related chronic DIC.

In summary, the incidence of IPNB is relatively low. Its main symptoms are abdominal pain, jaundice, and cholangitis, while other symptoms like anemia, thrombocytopenia, hypofibrinogenemia, and thrombosis are rare.^[2,3]^ We described the first report of above hematologic disorders presented in an IPNB patient. The treatment of IPNB is mainly surgery, and the prognosis is relatively good.^[3]^ However, clinicians should pay particular attention to the influence of IPNB on the hematological system and consider anticoagulation therapy and surgical treatment.

## Author contributions

**Conceptualization:** Ming Xiao, Aijun Sun, Canhong Xiang.

**Writing – original draft:** Ming Xiao.

**Writing – review & editing:** Ming Xiao, Aijun Sun, Fan Yu, Ying Xiao, Lihong Li, Dongyan Shen, Canhong Xiang, Jiahong Dong.
